# A Suite of Mobile Conversational Agents for Daily Stress Management (Popbots): Mixed Methods Exploratory Study

**DOI:** 10.2196/25294

**Published:** 2021-09-14

**Authors:** Matthew Louis Mauriello, Nantanick Tantivasadakarn, Marco Antonio Mora-Mendoza, Emmanuel Thierry Lincoln, Grace Hon, Parsa Nowruzi, Dorien Simon, Luke Hansen, Nathaniel H Goenawan, Joshua Kim, Nikhil Gowda, Dan Jurafsky, Pablo Enrique Paredes

**Affiliations:** 1 Department of Computer and Information Sciences University of Delaware Newark, DE United States; 2 Symbolic Systems Program School of Humanities and Sciences Stanford University Stanford, CA United States; 3 Computer Science Department College of Engineering Stanford University Stanford, CA United States; 4 Department of Information Systems Institut Supérieur d'électronique de Paris Paris France; 5 Stanford School of Medicine Stanford University Stanford, CA United States; 6 Alliance Innovation Lab Silicon Valley, CA United States; 7 Department of Linguistics School of Humanities and Sciences Stanford University Stanford, CA United States

**Keywords:** conversational agents, virtual agent, chatbot, therapy, stress relief, stress management, mental health, stress, exploratory, support, mobile phone

## Abstract

**Background:**

Approximately 60%-80% of the primary care visits have a psychological stress component, but only 3% of patients receive stress management advice during these visits. Given recent advances in natural language processing, there is renewed interest in mental health chatbots. Conversational agents that can understand a user’s problems and deliver advice that mitigates the effects of daily stress could be an effective public health tool. However, such systems are complex to build and costly to develop.

**Objective:**

To address these challenges, our aim is to develop and evaluate a fully automated mobile suite of shallow chatbots—we call them Popbots—that may serve as a new species of chatbots and further complement human assistance in an ecosystem of stress management support.

**Methods:**

After conducting an exploratory Wizard of Oz study (N=14) to evaluate the feasibility of a suite of multiple chatbots, we conducted a web-based study (N=47) to evaluate the implementation of our prototype. Each participant was randomly assigned to a different chatbot designed on the basis of a proven cognitive or behavioral intervention method. To measure the effectiveness of the chatbots, the participants’ stress levels were determined using self-reported psychometric evaluations (eg, web-based daily surveys and Patient Health Questionnaire-4). The participants in these studies were recruited through email and enrolled on the web, and some of them participated in follow-up interviews that were conducted in person or on the web (as necessary).

**Results:**

Of the 47 participants, 31 (66%) completed the main study. The findings suggest that the users viewed the conversations with our chatbots as helpful or at least neutral and came away with increasingly positive sentiment toward the use of chatbots for proactive stress management. Moreover, those users who used the system more often (ie, they had more than or equal to the median number of conversations) noted a decrease in depression symptoms compared with those who used the system less often based on a Wilcoxon signed-rank test (W=91.50; Z=−2.54; *P*=.01; *r*=0.47). The follow-up interviews with a subset of the participants indicated that half of the common daily stressors could be discussed with chatbots, potentially reducing the burden on human coping resources.

**Conclusions:**

Our work suggests that suites of shallow chatbots may offer benefits for both users and designers. As a result, this study’s contributions include the design and evaluation of a novel suite of shallow chatbots for daily stress management, a summary of benefits and challenges associated with random delivery of multiple conversational interventions, and design guidelines and directions for future research into similar systems, including authoring chatbot systems and artificial intelligence–enabled recommendation algorithms.

## Introduction

### Overview

In the United States, approximately 60%-80% of the primary care visits have a psychological stress component [1], but only 3% of patients receive stress management advice during these visits [2]. The reason for this is a combination of both limited infrastructure geared toward preventive health and limited focus on stress management. However, the increasing accessibility of mobile computing has spurred the growth of mental health apps, which currently account for 29% of the mobile health app market that includes fitness, nutrition, and other lifestyle apps [3]. However, general trends suggest that users are spending an increasing amount of time accessing services through messaging clients compared with purpose-built apps [3]. As a result, developers are leveraging these clients to build conversational interfaces, also known as *chatbots*, to create novel interactions in the health domain, including those that allow users to report symptoms, make appointments, and gain referrals.

Advances in natural language processing, such as intent [4] or emotional recognition [5,6] based on very large language data sets, continue to increase the range of these systems and their potential for impact. Research into improving conversational systems spans a number of domains such as customer service [7,8], companionship [9,10], and, increasingly, mental health [11-14]. As chatbots are scalable and easy to access, many systems are aimed at substituting human support in common conversations with known formats. Early efforts in mental health include ELIZA [15], which attempted to model the psychoanalytical approach of introspection: asking questions to engage the user in examining their own mental and emotional processes. More recently, chatbots such as Woebot [16] and Wysa [17] have been used to provide cognitive behavioral therapy (CBT) support to people at risk for depression. As a result, it is no surprise that a recent workplace survey found that most people (86% of those surveyed) were receptive to using chatbots and artificial intelligence (AI) systems that provide mental health support services [18]. However, given the complexity of life and the many types of stressors that a chatbot would need to understand to provide support, building a proactive everyday stress management chatbot is complex to design, costly to develop, and difficult to author in ways that appeal broadly.

To address these limitations, we aim to explore creating a new breed of simple conversational chatbots that use short conversations for in-the-moment management of daily stressors (eg, deadlines, difficult social interactions, and lack of sleep). Inspired by Etzioni’s second law for AI systems, “Disclose that it is not human” [19], we aim to create *shallow* yet effective and engaging mental health chatbots that do not try to replicate human intelligence. In the context of daily stress management, we define shallow chatbots as those that use few and brief conversational exchanges to deliver a single coping technique. These shallow chatbots are not created to replicate or replace humans (ie, family, friends, or therapists) but rather to operate as part of a larger ecosystem of agents providing stress management support. The advantages of creating multiple shallow chatbots are manifold: (1) chatbots capable of delivering microinterventions lower barriers of time and commitment for users; (2) they can be authored and curated more quickly by novice designers to produce a variety of high-quality advice options; (3) this variety of chatbots could help improve long-term engagement (ie, chatbots that *fail* could be removed); and (4) the suite approach allows for future personalization.

Prior research has explored the design of suites of just-in-time stress management interventions. For instance, the study by Paredes et al [20] demonstrated that a suite of microinterventions coupled with a web-based learning recommendation system could teach long-term stress coping skills to users. We extend this research on microinterventions by exploring a suite of diverse and specialized shallow chatbots for daily stress that we call *Popbots*. As early work investigating suites of shallow chatbots, our research questions are exploratory and include the following: *How might we design multiple shallow chatbots for proactive and reactive stress management? How might everyday users react to using these multiple chatbots for managing their daily stress? And what challenges and benefits do they perceive about such systems?*

### Background

#### Daily Stress

The stress response is an evolutionary mechanism that mobilizes bodily resources to help humans cope with daily challenges as well as life-threatening situations. Stress has two components: a stressor and a stress response. The former could be linked to sources of uncertainty, complexity, cognitive loads, or emotional distress. The latter refers to the mental and physical reaction to such stimuli. Daily stressors are defined as the routine challenges of day-to-day living. The challenges can either be predictable (eg, daily commutes) or unpredictable (eg, an unexpected work deadline) and occur on 40% of all days. Unlike chronic stress, these stressors are relatively short-lived and do not persist from day to day [21,22]. However, daily stress has been shown to exacerbate symptoms of existing physical health conditions [21]. Repeated triggering of daily stress can also lead to chronic stress, which has been associated with a variety of pathophysiological risks such as cardiovascular diseases and immune deficiencies—conditions that impair the quality of life and shorten life expectancy [23,24]. Thus, having effective mitigation strategies for daily stress can have a positive effect on a person’s well-being and overall health.

#### Traditional Stress-Mitigating Interventions

There is a wide variety of methods employed to help reduce stress. Positive psychology, for instance, is an emerging practice to help people calm down with personally targeted cues such as asking people to express gratitude or perform compassionate acts [25]. Another group of effective techniques is part of CBT [26], which teaches people how to recognize their sources of stress, change their negative behavioral reactions, and reframe their thoughts. Yet another approach is the use of narrative therapy, which focuses on constructing conversations to help people become satisfied with their state of being [27]. Such conversations are the basis of social interaction, which has a direct impact on emotions [28,29]. For example, positive social interactions have been shown to lead to calmness and openness in social engagement [29,30]. In our work, we borrow from this literature (ie, positive psychology, CBT, and somatic regulation) to design chatbots to guide users through stress-relieving techniques in response to daily stressors.

#### Stress-Mitigating Microinterventions

A relevant approach to this work is the use of internet-based technology that leverages specific aspects of CBT (eg, for smoking cessation [31,32]), positive psychology (eg, for depression [33,34]), and similar techniques to deliver personalized treatments and enhance well-being [35]. Recently, researchers explored the use of machine learning algorithms to recommend calming interactions with web apps. For instance, the study by Paredes et al [20] demonstrated the benefit of using just-in-time web-based interventions for teaching long-term stress-coping skills. In particular, the study discussed the complexity of engaging people to prevent early attrition. People under high levels of stress find that any additional task, including interventions, adds to their stress load. This motivates the need for research on the design of intervention suites that could reduce attrition by diversifying the types of interventions that are recommended to users over time [20,36,37].

#### Chatbots for Mental Health

Chatbots have a long history of application in mental health. The earliest mental health chatbot, ELIZA [15], was programmed to deliver nondirective therapy mirroring Rogerian therapy (ie, reflecting and rephrasing user input). A few years later, PARRY [38] was used to study schizophrenia. In addition to its capability of *displaying* regular expressions, PARRY included a model of its own mental and affect states. For example, PARRY could become more angry or mistrustful, thus generating *hostile* outputs. In a comparison study, psychiatrists could not distinguish transcripts of interviews with PARRY from those of interviews with people with schizophrenia. However, work on subsequent mental health chatbots did not emerge until recently [11-14].

Recent examples close to our work are varied and include chatbots that administer motivational stress management surveys [39] and CBT chatbots such as Woebot [16], Wysa [17], and Tess [40]. Woebot is an automated chatbot based on the principles of CBT. Woebot leads users through a series of CBT-type lessons, directing users to videos and other forms of didactic material to get them to engage in common CBT skills such as cognitive restructuring or behavioral activation. Wysa is an AI-driven *pocket penguin* that also bases chat interactions on CBT skills. The benefits of Woebot have been demonstrated in a randomized controlled trial showing superiority to a web-based e-book at reducing symptoms of depression and anxiety in a sample of college students, and a similar experiment was run with Tess, which corroborates these results across multiple university populations.

This expanding ecosystem of chatbots for mental health apps suggests that such tools are viable as accessible support solutions. This is not surprising, given that mental health has long relied on the *talking cure* as a primary form of treatment. A challenge regarding the use of existing chatbot systems is the need to explore the problems through a set of questions and answers and conversational exchanges that may be hard to author and maintain. Our system overcomes this limitation by allowing for the creation of multiple chatbots with each representing a single type of intervention. Authoring these *shallow* chatbots is easier for a designer because they can focus on delivering a single intervention technique with a clear objective and conclusion. For users, microintervention chatbots offer quick advice without their needing to work through a lengthy dialog that could be, by itself, another source of stress. In some ways, our system resembles a *game console* or a media platform (eg, Netflix) where each chatbot is a new *game* or *movie* and we can learn over time which chatbots the users prefer.

## Methods

### Prototype Chatbot Suite

**Figure 1:**
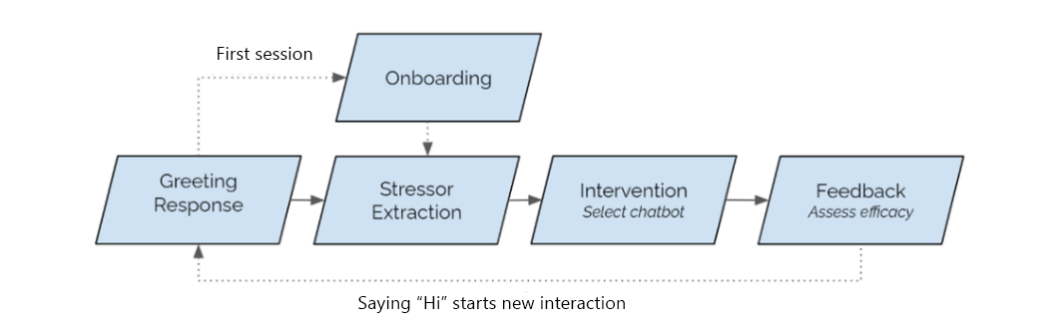
Overview of conversation structure for all chatbots: When a user sends a greeting message (eg, “Hi”), they receive a greeting from the suite of chatbots in response. If it is the user’s first time using the suite, they are directed to the onboarding script explaining how our shallow chatbot suite operates and what its limitations are. Next in the conversation sequence, the system asks the user what it is that is currently “stressing them out”; the stressor is then extracted, and a chatbot is randomly selected. Each chatbot delivers a coping technique in the form of a brief conversation that ends with the user assessing the conversation on a 3-point Likert scale (ie, Not helpful, Neutral, and Helpful).

Extending prior work on microinterventions and conversational interfaces [20], we propose the creation of a suite of shallow chatbots that provide in-the-moment conversations for managing daily stress. Although prior work tended to focus on patients or people at risk (ie, people with high levels of depression or anxiety symptoms as highlighted by recent surveys [11-14]), our aim is to provide a quick and engaging system using simple microintervention chatbots that can help to alleviate daily stress for healthy people (ie, toward improving long-term well-being and helping to mitigate future crises). Another goal of the project is to simplify the authoring of chatbots by reducing complexity toward enabling a scalable solution for rapidly creating numerous (ie, hundreds or more) chatbots for stress management. To explore this idea, we developed a prototype chatbot suite with a common template for short conversations (ie, 2-3 minutes with a few conversational exchanges) composed of four components (Figure 1): (1) an onboarding script for explaining the system and its limitations to users; (2) a shared set of greetings, stressor parsers, and intent-extraction components; (3) the microintervention chatbots that make up the suite; and (4) a feedback component.

### Chatbot Design

#### Overview

We used an iterative, human-centered approach to designing our chatbot suite (Table 1). The initial chatbot scripts were developed in a 4-hour workshop with the aid of 6 novice designers, curated by a clinical psychologist, and tested for quality purposes by conducting simulations in which pairs of designers acted as users and chatbots. Each chatbot relied on a decision tree to facilitate conversations, usually resulting in the user providing a response to a series of open-ended (eg, *What is the worst-case scenario for [a stressor]?*), yes-no (eg, *Has [the stressor] affected your sleep?*), or numerical (eg, *What is the severity of a scenario?*) questions (Textbox 1). Stress management literature—particularly literature related to CBT techniques [26,41,42]—was used to derive conversations for stress relief. Using this approach, our novice design team created chatbots based on three techniques (ie, worst-case scenario, problem solving, and positive thinking). The total development time (ie, including design, curation, and quality assurance steps) was approximately 8 hours. We then evaluated the feasibility of our chatbot system against a control condition in a Wizard of Oz (WOZ) pilot study with 14 users (Multimedia Appendix 1). We observed that the participants in the condition with multiple chatbots tended to agree to a greater degree that the intervention helped to reduce their stress compared with those in the control condition with a single chatbot; however, follow-up interviews revealed that the participants still expected chatbots to act in human-like ways. The lessons learned from this pilot study were used to refine our chatbot scripts, and they also informed the development and implementation of our web-based system.

**Table 1:** Prototype chatbot names, their techniques, and the studies in which they were used.

Chatbot	Technique	Description	Study
Doom Bot	Worst-case scenario	Asks the user to consider the worst-case scenario	Wizard of Oz and web-based
Sherlock Bot	Problem solving	Asks a series of questions to pinpoint the problem	Wizard of Oz and web-based
Glass-Half-Full Bot	Positive thinking	Asks the user to view their problems in a new light	Wizard of Oz and web-based
Sir Laughs-a-Bot	Humor	Finds humor in the situation	Web-based
Treat Yourself Bot	Self-love	Reminds the user that it is all right to treat themselves	Web-based
Dunno Bot	Distraction	Asks user to think about events they are looking forward to	Web-based
Checkin Bot	Checking in	Asks whether the stressor affected daily activities	Web-based

**A sample chatbot script.**

**The script used by Doom Bot**

- Tell me more details about [problem]?
- I’m sorry to hear that. What are you most afraid might happen as a result?
- Alright, on a scale of 1 to 10, 1 being impossible, 10 being certain, how likely is this scenario?
- Alright, in the case that this happens, what could you do to get back on track?
- Cool, looks like you have a plan B. Just remember, though you cannot control everything, there is a way to get back on your feet.

#### System Implementation

**Figure 2:**
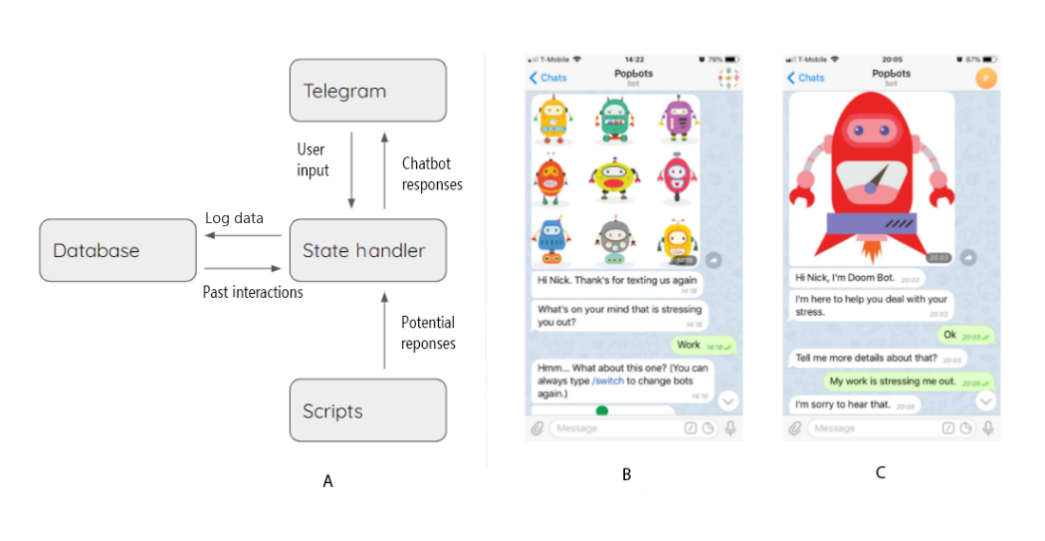
A system diagram overview and example conversation scripts with conversational interfaces: (A) System diagram; (B) User who initiated a conversation over the Telegram interface being asked to describe their stressor; (C) Sample conversation with Doom Bot, recommended by the system.

We implemented our chatbot suite in Telegram (Telegram Messenger Inc) [43], a data-security–compliant messaging platform, using a Python (Python Software Foundation) backend and a MongoDB (MongoDB Inc) database (Figure 2). Using prior experience and the observations obtained during the initial chatbot workshop, the research team generated 4 additional chatbots bringing the total to 7 and programmed the conversational scripts in Python. Interactions with these chatbots are automatic, rely on open text (as opposed to buttons), and are rule-based, using regular expressions to control the flow of conversations. Following our template, when the user messages the chatbots (ie, by typing “Hi”), they receive a friendly greeting message and are asked to describe their current stressor (Figure 2). After extracting the stressor, a chatbot is randomly recommended, and its avatar image is displayed (Figure 2). User input is passed to a state handler through the Telegram application programming interface; the state handler analyzes these data to generate a response. Once the response is generated, it is sent to the user, and the interaction is logged. After the conversation ends, the chatbot thanks the user for sharing and asks them for feedback on whether the interaction helped to reduce their stress on a 3-point Likert scale (ie, *Not helpful*, *Neutral*, and *Helpful*). We refined the chatbots with pilot users to make them seem more human-like (eg, introducing typing delays), clarified utterances so that users were more aware of when the system was waiting for input, and added a */switch* option that allows users to change chatbots in situ (the only interaction that used buttons). Sample conversations are included in Multimedia Appendix 1.

#### Protocol

The participants were recruited in August-September 2019 through word of mouth and a university listserv. Our recruitment materials specified that participants would be asked to use our system for 7 days and complete a prestudy questionnaire, short daily surveys, and a poststudy questionnaire. These materials also specified that participants must be aged 18 years or older and have a compatible smartphone (ie, an Android [Google LLC] phone or an iPhone [Apple Inc]). Web-based enrollment occurred on a rolling basis, and all questionnaires were completed through the Qualtrics survey tool (Qualtrics LLC).

After receiving our invitation email, the participants completed our prestudy questionnaire that asked them about their demographic information, how much stress they felt daily, and their perceptions of using chatbots for daily stress management. The participants also completed the short Patient Health Questionnaire (PHQ)-4 to ascertain a measure of their clinical anxiety and depression symptoms [44]. Upon completing the survey, the participants were automatically sent email instructions for installing the Telegram app as well as a personalized URL that, when accessed on their smartphones, initialized the *Popbots*channel within the app.

Once this initialization was completed, the participants were instructed to type “Hi” and go through the onboarding script that explained the purpose of the system (eg, that it was for daily stress management) and its limitations (eg, that it was not intended for the treatment of serious mental health conditions). After going through the onboarding script, the participants were instructed to interact with the chatbots anytime they felt stressed over the next 7 days. Daily surveys, which were sent at 8 PM each day (local time), asked the participants to rate their daily stress levels, sleep quality the previous night, and level of social interaction experienced that day. After 7 days of using the system, the participants completed a poststudy questionnaire, which asked the participants about their perceptions of daily stress over the course of the week and if their perceptions of chatbots had changed, as well as other usability questions. The participants also completed the PHQ-4 questionnaire again. We then followed up with a subset of the participants to complete a semistructured interview and card-sorting task (similar to our pilot WOZ study); we sent a general email request to all participants, and volunteers were enrolled on a first-come, first-served basis.

To motivate participation, we provided compensation. The participants earned US $10 through an Amazon gift card (Amazon Inc) for successfully completing both the pre- and poststudy questionnaires. We offered an additional US $3 for each day that they interacted with the chatbots and completed the daily survey. Compensation was prorated based on partial completion of these components. The participants who were interviewed after the study were compensated with an additional US $25 per hour of the interview. The protocols were reviewed for ethics and privacy concerns by our institution’s research compliance office.

#### Participants

We recruited 47 participants (34 women and 13 men). Most (33/47, 70%) were university staff members, whereas the remaining participants (n=14) were undergraduate students. Although the staff members were aged between 18 and 74 years, the students were aged between 18 and 24 years (Table 2). Approximately half of the participants (21/47, 45%) identified themselves as Asian, whereas the remaining participants identified as White (n=12), Hispanic or Latino (n=7), Multiracial (n=2), Black (n=2), American Indian (n=1), or preferred not to identify their race (n=2). More than half (28/47, 60%) reported being single (with no children), less than half (n=18) were married or in a domestic partnership (mean 1.9 children), and 1 participant was separated (with 3 children). Although the students had completed high school or General Educational Development requirements and were now working on their bachelor’s degree, the staff members had a high degree of formal education, with more than a third holding a bachelor’s degree (13/33, 39%), just less than a third (n=9) holding a master’s degree, some (n=4) having some college course experience, and a few more (n=4) holding terminal professional or doctoral degrees. Most of the staff members (30/34, 88%) were employed full time, whereas the remaining (n=4) were working part time; all student participants (14/47, 30%) listed their occupation as full-time students. Excluding interview payments, the participants received an average of US $23.86 (SD US $14.35; median US $25.00) in compensation.

**Table 2:** Participant age ranges by subpopulation.

Population	18-24 years, n (%)	25-34 years, n (%)	35-44 years, n (%)	45-54 years, n (%)	55-64 years, n (%)	65-74 years, n (%)
Students (n=14)	14 (100)	0 (0)	0 (0)	0 (0)	0 (0)	0 (0)
Staff members (n=33)	5 (15)	6 (19)	9 (26)	6 (19)	5 (15)	2 (7)

#### Data and Analysis

In summary, our data include responses to pre-, daily, and poststudy questionnaires; application logs from the chatbot system; interview transcripts; and photographs of the assignments made during the card-sorting activity. All questionnaires include Likert scale questions and short open-form responses. The follow-up interviews were audio recorded, transcribed, and coded for themes of interest. We pursued an iterative analysis approach using a mixture of inductive and deductive codes [45]. We created a codebook initially derived from research studies, our study protocol, and postinterview discussions among the research team members. The unit of analysis was the answer (or stream of answers) to specific questions. High-level codes included perceptions of chatbots for stress management and preferences regarding conversational partners, as well as privacy and trust. A random transcript was selected and cocoded by the research team members. The remaining transcripts were divided and coded independently. The individually coded transcripts were then reviewed by a second researcher who met with the original coder to resolve disagreements. In all, 2 researchers then aggregated the transcripts, reviewed them for consistency, and summarized the results.

Although 47 participants enrolled in the study, 31 (66%) completed both the pre- and poststudy questionnaires. As exploratory work, we report on descriptive statistics such as means and SD, which are contextualized with participant quotes. We use the letter *P* and randomized IDs to refer to the participants in our web-based study (eg, P1234) and letters (eg, PX) to refer to those from our WOZ pilot study in our interview results.

## Results

### Application Logs

Over the course of 7 days, most of the participants (44/47, 94%) interacted with our chatbots, generating 291 conversations. The participants averaged approximately seven conversations per week (mean 6.83, SD 3.14). These conversations were short, lasting only a few minutes (mean 1.95, SD 2.53), and often occurred during the latter part of the day. Although some conversations were likely triggered by the daily survey reminder (at 8 PM), most (232/291, 79.7%) of the conversations were unprompted and occurred throughout the day with increased activity in the 7 AM, 12 PM, 3 PM, and 8 PM hours. A deeper exploration of these conversations indicated that some participants were simply checking in, particularly around 8 PM, reporting stressors such as “Nothing” or “Doing pretty good actually.” As a result, we filtered out approximately a third of the conversations that fell into this category as well as those that contained a technical issue making them indecipherable.

### Reporting Stressors

We observed 2 ways that the participants reported stressors to the chatbots. Most of the participants (146/197, 74.1% of conversations) tended to describe stressors in a few words. For example, participants wrote “Having to go to work tomorrow,” “My presentation that’s coming up,” and “My friend being mad at me.” Another approach (51/197, 25.8% of conversations) was to type out single words (eg, “Money,” “Car,” and “Family”).

### Topics of Conversation

After filtering out erroneous and nonstress-related conversations, we labeled the remaining 197 conversations using eight category tags representing the consistent topics that the participants discussed with the chatbots (Table 3). The most common topics included (1) work- and school-related productivity issues, (2) health problems (eg, feeling tired and experiencing pain), and (3) interpersonal issues related to (nonfamilial) social relationships. There were also a number of *Other* conversations that were not widely discussed but might point to additional topics of daily stress, including vacation-related stress (eg, packing), commuting, and seasonal stressors (eg, holiday-related gift giving).

**Table 3:** Categories of stressors.

Stressors	Examples	Count (n=197), n (%)
Work, school, and productivity	“I have some tasks I keep putting off”	79 (40.1)
Health, fatigue, and physical pain	“I want to eat better but I’m having a hard time with it”	27 (13.7)
Social relationships	“I found out my ex has a new girlfriend”	21 (10.6)
Financial problems	“I have a friend coming by and I’m stressed about an expense”	13 (6.6)
Emotional turmoil	“Feeling lonely”	12 (6.1)
Family issues	“My marriage”	10 (5.1)
Everyday decision-making	“Don’t know what to cook for dinner”	8 (4.1)
Other	“Just travel stuff”	27 (13.7)

### In Situ Efficacy

Overall, in situ efficacy was either helpful (76/197, 38.6%) or neutral (64/197, 32.5%). As it is reasonable to expect that not all interventions will be viewed as helpful, a neutral response may also be viewed as positive in terms of a system with multiple chatbots. However, more concerning is that the remaining conversations were rated as unhelpful (57/197, 28.9%). We also observed that feedback varied by chatbot (Figure S1 of Multimedia Appendix 1). For example, nearly half of the conversations of Treat Yourself Bot were rated as helpful and most (22/31, 71%) were rated positive or neutral versus those of Checkin Bot, which were mostly viewed as unhelpful (13/25, 52%). We believe that these results are encouraging because they suggest that with more data, patterns between stressors and chatbot or user and chatbots may emerge that might explain these differences and allow a future system with more complex recommendation algorithms to learn from and make personalized recommendations for each user-stressor pair.

### Daily Surveys

The daily survey was administered each day at 8 PM (local time). In addition to usability questions, the survey tracked levels of stress, social interaction, and sleep quality the previous night using 5-point Likert scales ranging from *None* to *Very high* or *Very poor* to *Very good*. As a third (67/197, 34%) of the conversations could not be matched to a daily survey (ie, because the participants did not complete them that day), our analysis focused on evaluating trends in the matched conversations (n=130). With respect to general trends, we noted that most (91/130, 70% of conversations) were matched to surveys that reported *Low* to *Moderate* levels of stress throughout the week. Similarly, most (114/130, 87.6%) were matched to surveys reporting *Acceptable* or better sleep quality the night before and most (110/130, 84.6%) to surveys reporting *Low* to *High* levels of social interaction each day. However, conversational feedback was roughly evenly distributed across these variables, skewing slightly toward *Helpful*. Further analysis did not reveal any strong correlations between these variables and in situ feedback.

### Poststudy Experiential Feedback

Open-ended feedback from the poststudy questionnaire was generally positive and helped to characterize the participant experience. For example, although it seemed from the application logs that the participants were using the chatbots throughout the day, most considered using the chatbots to be a private activity and, as a result, reported that they were difficult to use in the moment. Most of the participants (28/31, 90%) reported using the chatbots when they were alone—typically when they had a free moment (ie, a few hours after a stressful event). This was often because in their work and social environments, they were busy or wanted to avoid giving the perception of rudeness caused by being on their phones, which is an interesting potential barrier.

Like the in situ conversational feedback, retrospective feedback on effectiveness skewed positive. Most (25/31, 81%) of the participants viewed the chatbots as *Slightly effective* to *Very effective*, and approximately one-fourth (9/31, 29%) described the chatbots as *Not effective at all*. Approximately half (17/31, 55%) of the participants described the current set of chatbots as cute and engaging. They also appreciated the concept of having a variety of chatbot options available. A participant explained as follows:

I like the ability to have access to different chatbots. I liked problem solving bot and Checkin-bot, but the laugh bot not so much.

P7596

However, with only 7 chatbots available, some (n=6) of the participants commented that their interactions with the chatbots felt formulaic or repetitive.

### Pre- and Poststudy Comparison

#### Post Hoc Analysis

As part of our analysis, we looked at changes in several questions asked across the pre- and poststudy questionnaires. These pre/post metrics include changes in the PHQ-4 scores, perceptions of daily stress, and perceptions of chatbots for stress management. To further explore these differences, we also conducted a post hoc analysis. We separated users into 2 groups based on the number of conversations that the participants had had with the chatbots. Specifically, we grouped participants whose completed number of conversations was less than or equal to the median number of conversations into the *Low use* group and the remaining into the *High use* group. The participants in the *Low use* group (n=16) had an average of 4.31 conversations over the course of the week (SD 1.31), whereas the participants in the *High use* group (n=15) had twice as many conversations (mean 8.67, SD 2.12).

#### PHQ-4 Scores

Overall, we observed a decrease in PHQ-4 scores over the course of the week when comparing pre- and poststudy assessments for the participants who completed the study (Figure S2 of Multimedia Appendix 1). The medians of the before-and-after PHQ-4 scores were 3.0 and 2.0, respectively. A Wilcoxon signed-rank test showed that this decrease was significant (W=91.50; Z=−2.54; *P*=.01; *r*=0.47). Although we cannot directly attribute this decrease to the interactions with our chatbots without a control group, our post hoc analysis suggests that although the scores of both groups for the prestudy PHQ-4 were similar (median 3.0), there was a greater reduction in the PHQ-4 score (median 2.0) of the *High use* group, which was significant (*W*=18.0; *Z*=-2.16; *P*=.03; *r*=0.57), compared with that of the *Low use* group (median 2.5), which was not. We theorize that these data point to the potential efficacy of our approach.

#### Daily Stress Experience

We evaluated perceptions of daily stress using a 4-point Likert scale ranging from *A little* to *A great deal*. Although the participants reported varying levels of stress on the daily survey, most described their perceptions of daily stress as *Moderate* in the prestudy questionnaire, and the perceptions of daily stress after their participation were retrospectively similar (Figure S3 of Multimedia Appendix 1). Although we observed a slight decrease in perceived daily stress, these changes were not significant.

#### Perceptions of Chatbots

When asked to describe their perceptions of chatbots for stress management on an open-response question, approximately half of the participants (22/47, 47%) were neutral (ie, they stated that they had no opinion on chatbots); slightly more than a third (n=17) were positive (ie, they believed that chatbots could be helpful); and the remaining (n=8) participants were negative (ie, they believed that chatbots would not be effective). An illustrative comment in favor of chatbots was by P8530: “They seem to be a viable option for the management of stress, but they need to be further refined in order to be useful in day-to-day situations.” In contrast, those who were more negative were best exemplified by P5219, who wrote: “...it doesn’t seem like talking to a non-human would be that helpful because, for me, talking to a human doesn’t usually help.”

However, in the poststudy questionnaire, most (20/31, 65%) of the participants reported a more positive attitude toward chatbots for mental health. This was often because (1) they had a positive experience with the system themselves, (2) they could conceive of such systems being helpful to people more generally, or (3) they found the activity of taking some time out each day to think about their stress helpful. In addition, approximately half (16/31, 52%) of the participants agreed that they had learned something about stress management from interacting with the system. For example, P8002 noted, “I liked the idea of congratulating yourself for the things you did manage to do rather than focusing only on what you didn’t.” Interestingly, even the participants who did not report learning anything from the system were positive. For example, P9907 noted that although they did not learn anything from the interactions with the chatbots, they were “helpful reminders of what I should be doing when I am stressed.” Others noted that although they did not learn anything directly from the chatbots, they did learn that chatbots could be effective tools. A third of the participants (n=10) reported no change in their general attitudes toward chatbots, and a small number (n=3) reported a more negative attitude (ie, they found the chatbots too repetitive or poorly implemented).

### Follow-up Card-Sorting Interviews

#### Two Phases

The interviews primarily centered around a card-sorting activity with two phases. In the first phase, the participants (N=13) were given 13 stressors to be assigned to the different chatbots based on the chatbots that they felt were most effective. The stressors were synthesized from the Holmes and Rahe Stress Scale [46]. In the second phase of the activity, the participants were asked to redistribute the stressor categories among three additional human options in addition to the chatbots: a nontrained stranger, friends and family, and a therapist. The participants were asked to *think aloud* while making their assignments.

#### Card-Sorting Results

The card-sorting activity suggested that there were certain stressors that the participants preferred to talk to the chatbots about, given that not all stressor categories were reassigned in phase 2 when humans were available. We observed that 47% (79/169) of the stressors were retained by the chatbots (Figure S4 of Multimedia Appendix 1). This result, we believe, is critical and points toward a willingness by the participants to use the chatbots for common daily stressors.

Moreover, when we sort these stressors by those most assigned to the chatbots, we observe that *Everyday decisions* and *Financial stress* were rarely reassigned to humans, whereas interpersonal issues such as *Romantic stress* or *Conflict with family* and complex topics such as *Sexuality and identity* were. However, not all chatbots performed equally well in terms of retaining their assignments in the presence of humans. For example, Table 4 indicates that Checkin Bot, Sherlock Bot, and Doom Bot were some of the more resilient chatbots, whereas most of Dunno Bot’s assignments were reassigned to humans. In fact, many chatbots retained more than half of their assignments. We also noted that the participants had a strong preference for assigning problems to *Friends and family* over *therapists,* with two assignments made to strangers.

**Table 4:** Stressor assignments by chatbot and human resource (n=169).

Resource: chatbots and humans	Stressor assignments, n (%)	
	Phase 1: chatbots	Phase 2: chatbots and humans
Sherlock Bot	45 (26.6)	21 *(12.4)*^a^
Glass-Half-Full Bot	30 (17.7)	5 *(2.9)*
Doom Bot	23 (14)	12 *(7.1)*
Sir Laughs-a-Bot	21 (13.6)	12 *(7.1)*
Treat Yourself Bot	20 (11.8)	11 *(6.5)*
Dunno Bot	15 (8.9)	5 *(2.9)*
Checkin Bot	15 (8.9)	14 *(8.3)*
Friends and family	N/A^b^	59 (34.9)
Therapist	N/A	29 (17.2)
Stranger	N/A	2 (1.2)

^a^Values in italics indicate that the percentage of the total decreased compared with phase 1 when human resources were unavailable.

^b^N/A: not applicable.

#### Qualitative Insights

##### Important Themes

As the participants made their assignments of stressors to the available chatbots and human resources, we probed for their rationale. Overall, we corroborated important themes around the desire to have chatbots that are part of an ecosystem of support supplementing humans, behave in a human-like way, and are available to discuss certain stressors.

##### First Impressions

A challenge with chatbots is that of first impressions. Approximately half of the participants (6/13, 46%) thought that their first interaction with a chatbot had an impact on their overall perceptions of the multiple chatbots available, and an unpleasant first interaction with a chatbot left the participants with a negative impression. For example, 1 participant stated as follows:

I went on the app and the bot said, “Find a joke” and it was something actually really terrible that was going on. That was my first time interacting with the bots. I thought “Wow, there’s nothing that’s funny about this.” This is not helpful at all.

P1962

##### Benefits of Multiple Chatbots

The participants described several benefits of having multiple chatbots available, including the ability to combine two or more chatbots to address a problem. This point was raised during our WOZ experiment by PB, who was insistent that problem solving is ultimately the solution to all stressors, although other interventions may be used before, or in conjunction with, problem solving for better results:

Everything is going to end up here in problem solving. If people are calm and collected, then they can think well. So, if people are calm first, then they will find everything’s fine. Sometimes you can go from extreme stress to humor, but that’s a big jump. I think it’s better if you’re slightly calmer and then humor comes in and then distraction.

PB

We probed this idea further during the latter phases of our web-based study. Nearly half (6/13, 46%) of the participants agreed that using chatbots (or interventions) in combination could be an effective strategy to address stressors. For example, several were interested in using other interventions in combination with problem solving:

In the case of conflict with a coworker, distracting yourself, not letting it take over your life, looking at the positive side of things could help. It could also go to the treat yourself. And then the worst-case scenario, ‘Sure, I no longer interact with this coworker, and that’s okay.’ In the end going back to the Problem Solving.

P5279

However, 1 participant (P5981) noted that while interacting with more than 1 chatbot can be helpful to address a problem, it is not necessary to use them in rapid succession.

##### Talking With Friends and Family

Most of the participants (11/13, 85%) favored talking with friends and family over talking with chatbots in some cases, and they indicated that this preference had to do with the complexity of the stressor. The participants preferred speaking with friends and family about difficult emotional problems (eg, conflicts with coworkers or interpersonal relationships). A participant summarized as follows:

It depends on the degree of the problem. If it is a huge problem, I want a real person. If it’s medium to small problem, then I go to the bot.

P1442

There were several reasons for this preference, including relationship history and range of responses. Friends and family already have pre-existing relationships with the participants and knowledge about their personal lives. Approximately a third (n=4) of the participants preferred humans because they can show empathy. Another third (n=4) believed that humans are better at problem solving.

##### Talking With Therapists

Similar to talking with friends and family, more than half of the participants (7/13, 54%) said that they believed that therapists would be more helpful than chatbots in resolving complex problems. A participant observed as follows:

Therapists are trained and objective. They are actual people. You can have complex conversations and get answers to questions with them.

PC

For example, nearly half (3/7, 43%) of the participants believed that a therapist would be very helpful for talking through issues of sexual identity.

##### Talking With Chatbots

The participants noted several practical and emotional benefits of talking with chatbots. Regarding practical reasons, most (11/13, 85%) of the participants suggested that chatbots have some advantages over humans. Almost half (n=5) of the participants mentioned that talking to a chatbot could help them avoid putting an undue burden on others. A participant stated as follows:

[Work stress] can be in the middle of the day, and [my friends] are going to be busy, and I don’t want to text them and bother them about that

P7596

Similarly, some (n=3) of the participants also noted that chatbots are easy to access:

It’s going to be a lot quicker to pull up an app, right? I sneak away to a room, I pull up the bot app, it’s a lot quicker than messaging someone like, ‘Hey, are you around?’ and then waiting for a message back, or calling someone.

P7596

Another reason cited by a few (n=3) of the participants was that they could more easily control how much they told chatbots, whereas humans are more likely to press for information.

Regarding emotional coping, the participants explained that the chatbots allowed them to shift their thinking about their stressors. For example, more than half of the participants (8/13, 62%) reported that Doom Bot helped them to recalibrate the gravity of their stressor:

It’s nice to hear when it feels like you’re on the brink of doom, that like, oh, this is the worst thing that can happen.

P5279

Other participants (n=4) mentioned Glass-Half-Full Bot as being effective for putting stressful events in a different light. A participant, PD, shared that reflecting on the positive aspects of their experience allowed them to *“*take the edge off and make [the situation] work.” Similarly, half (n=7) of the participants described the chatbots as a distraction from their problems, potentially because of the immersive nature of conversational interventions. Moreover, almost half of the participants observed that humor helps them ameliorate their stress; as 1 participant, P7616, stated, “Humor is often the antidote.” These participants noted that chatbots with amusing dialog could be especially effective for stress management, although humor is highly subjective and thus difficult to make sure it appeals to everyone.

##### Privacy and Trust

When the participants were asked about any privacy concerns that they had about the platform, they were split. Approximately half (6/13, 46%) of them found some topics too personal to discuss with friends and family, but they were open to talking to chatbots because of the perceived privacy they provide. For example, a participant noted as follows:

I’m a very private person. I don’t like to talk about a lot of things even with friends and family or in therapy.

P1962

Others went so far as to say that chatbots were more trustworthy because, as P7596 stated, they are *“*devoid of things that come with being human-like judgment or telling secrets.” In contrast, a few (n=4) of the participants noted that they were aware that their messages were not private and took comfort in knowing that therapists were ethically bound to keep conversations confidential. The remaining participants (n=3) were unsure:

I don’t know whether to worry about privacy or not. I think I have brand loyalty, so I always feel like Apple is gonna keep my stuff private.

PD

When time allowed, we probed a bit more on this topic to get a sense of how users felt about chatbot systems using their data to improve the systems’ usefulness, and 2 concerns emerged. First, approximately a third (4/13, 31%) of the participants expressed concern about the use of conversational logs and other metadata that can be collected about web-based experiences. For example, P1962 likened such systems to other technology-related privacy incidents, stating, “even though I found the chatbots helpful, if they were like [Amazon’s] Alexa, running in the background waiting and listening to you and recording everything, I wouldn’t like that.” One-fourth (n=3) of the participants were concerned that, even with additional training, chatbots might not be able to be trusted to handle mental health crises (eg, referring users to proper resources). As P6716 summarized, “Chatbots should potentially set off an alarm and say there needs to be a human to prevent this person from doing something terrible, as opposed to just being an ultra-safe communication cocoon.” In contrast, 2 participants were unconcerned about the handling of their data as long as it was used to improve their experience. As P5219 stated, “I’m okay with chatbots having a lot of data about me if it’s going to help them to respond better.”

## Discussion

### Principal Findings

In this work, we explored the potential effectiveness and user perceptions of a suite of multiple chatbots for the management of daily stress in a web-based study. Our results suggest that multiple shallow chatbots, grounded in CBT and other techniques, can be designed quickly by relatively novice designers and that these chatbots could have a positive impact on mental health and well-being. We draw these conclusions from the observations that the in situ feedback indicated that most conversations were viewed as helpful, or at least neutral, and that there was a reduction in the PHQ-4 scores. As a complement to these results, there was the general positive improvement in sentiment toward the effectiveness of chatbots for daily stress management as well as other qualitative feedback that was consistent with these conclusions. However, participant bias is a concern when evaluating this feedback.

Although this study lacked a direct control, the fact that there seemed to be a bigger reduction in the PHQ-4 scores in those who used the system more often is encouraging, given that the users also perceived the level of daily stress that they experienced during participation as consistent with their prestudy perceptions of daily stress. Although we did not observe a reduction in these retrospective perceptions of daily stress, it is unlikely that such perceptions would shift, given the duration of the study and the general health of the population (ie, most of the participants reported sleeping well, being social, etc). Moreover, although many participants were positive about the variety of the chatbots in our suite, some indicated that it was not necessarily the conversations that they had had that were helpful but rather the act of taking the time out to reflect. Although we generally make no distinction between a chatbot in our system and a microintervention, the act of taking some time out is itself a microintervention, regardless of the user’s feedback on the chatbot that they were paired with. From the perspective of our system, either outcome is acceptable if users are engaged with the system and this engagement results in users being better equipped to manage daily stress. Next, we discuss some additional observations and opportunities enabled by our work, as well as its limitations. We close with design recommendations and discuss areas of future work.

### Ecosystems of Support

When the participants were asked to assign the stressor categories to available human and chatbot resources in our follow-up card-sorting tasks, it was interesting to observe that nearly half of the daily stressors assigned to the chatbots remained with them when humans are also available. This suggests that, in the context of proactive stress management, the participants viewed chatbot systems such as ours as expanding the ecosystem of available support. This observation alone is critical because it suggests the potential of our system, and of chatbots more generally, to help with proactive stress management. The impact of a successful implementation could greatly increase access to stress management advice with a potential downstream impact of improving users’ well-being and helping to mitigate future crises. When the participants were asked to explain the rationale behind these assignments, they stated that they viewed chatbots as most effective for coping with low-complexity stressors (ie, practical and day-to-day concerns) compared with high-complexity stressors (ie, those of a more social or interpersonal nature) because of the relative ease of accessing chatbots and the perception of privacy granted by such systems. Another benefit that the participants perceived about their experience with our suite of chatbots was the potential to reduce the burden on available human-provided coping resources, which was also observed with other mental health chatbots used in long-term care [47].

### Lowering Barriers to Authoring Chatbots

The participants who completed the study were positive about their use of the chatbot suite. Some benefited by learning new coping techniques (eg, positive reframing) and others by being reminded to take a moment out of their day to reflect. Although our results are preliminary, we believe that this interest in using a variety of chatbots for different problems or using multiple chatbots in sequence (as reported by some users) could improve engagement and help prevent attrition in chatbot systems for mental health—a problem observed in recent studies [48]. Although our implementation was also faced with these problems, a key difference in terms of solutions is that authoring new content in our suite means simply authoring another shallow chatbot, which can be done rapidly, whereas authoring new content for a single chatbot system must be done in a way that matches existing traits (eg, personality) and norms, which can be a limiting factor. The suite approach, we believe, sets an expectation of new and different content, decreasing this burden on design and offering more opportunities to appeal to different users. This unique solution could reduce the complexity and cost of developing chatbots for mental health by shifting focus to simpler chatbot designs. However, our work also suggests that relatively simple chatbots that disclose that they are not human should still *appear* human and converse in human-like ways (eg, show empathy [49]) if they are to be accepted and engaging.

If future controlled and longitudinal studies demonstrate that suites of shallow chatbots can be effective, then another long-term benefit of this approach could be the democratization of chatbot design, which is dominated by professionals who are highly trained in user experience, linguistics, and other fields. In contrast to the narrative that experts know best, in *Democratizing Innovation*, Von Hippel [50] argues that users generate significant design innovations more effectively than experts because they are highly motivated to solve their problems and share solutions. If we can design tools and methods that make authoring chatbots easier (eg, reducing the need to understand complex linguistic topics), then we can greatly reduce barriers to authoring effective chatbots in both multiple- and single-chatbot scenarios. We envision a future where anyone from everyday users to clinicians looking to augment or supplement their practice can author and recommend shallow chatbots to others as an immediate coping resource for daily stress. However, this raises the question of challenges that need to be addressed, including reducing the need to learn complex conversational design tools.

### Recommendation Systems

In this study, chatbots were recommended to users at random, but an alternative approach could be the use of a recommendation system. Although conversational feedback skewed positive, some chatbots performed better than others (ie, feedback was more positive), and we theorize that installing a reinforcement learning algorithm that can better match a shallow chatbot to the user’s stressor could improve feedback further. For example, we noticed in some of our conversational logs that Doom Bot, which asks users to think about a future worst-case scenario, is not always appropriate for dealing with problems that exist in the past. Moreover, as our collection of shallow chatbots increases, it will be almost impossible for users to select an appropriate chatbot themselves, given the potential multitude of chatbots enabled by our less cost-intensive authoring paradigm. Similar to the study by Paredes et al [20], such algorithms can better take into account contextual, conversational, and prior interaction data to improve the matching between user problem and shallow chatbot, potentially personalizing to the user’s specific preferences over time. Chatbots that perform well across users could help solve the first-impressions challenge raised by our participants, which is an interesting solution not afforded to single chatbot systems [8]. In contrast, chatbots that generally perform poorly will not be recommended and thus could be discarded. In the future system we are working toward, users would be able to author many shallow chatbots quickly and deploy them, and the recommendation system would play an important dual role: recommending and curating appropriate and efficacious shallow chatbots that fit user context and stressor.

### Ethical and Privacy Considerations

In our follow-up interviews, the participants raised concerns related to privacy and ethical responsibilities, which our system shares with other chatbot systems. As has been observed in recent studies, this includes preserving user privacy, detecting what problems the system can handle and when escalation to a human is necessary, and clearly describing the limitations of the system and ensuring that such systems are safe to use [51,52]. Although we did not necessarily discuss this topic with all participants, we learned that participants vary in terms of their understanding of, and preferences toward, privacy. Some trusted the system to remain private, whereas others knew that researchers and developers would use these data to make improvements. Still others pointed out that the chatbots allowed them to control the amount of information that they needed to divulge to mitigate their stress, which was appealing and certainly suggests that detecting something like escalating a problem could be quite challenging when less detailed information is being provided. This last point, we believe, is interesting because users do have an agency, which should be respected, but it is clear from other domains that explaining permissions and limitations of web-based systems is a challenging topic to get right [53]. As our system grows, we will increasingly need to accommodate differing privacy preferences and levels of agency with respect to important concerns such as user safety.

### Design Recommendations

On the basis of this study, researchers and app designers engaged in designing multiple chatbots with a similar architecture might benefit from considering the following design recommendations:

1. Focus on lowering barriers to authorship and generating numerous shallow chatbots based on the vast amount of available psychological interventions for stress management.
2. Design for learning algorithms to handle recommendation and curation of interventions.
3. Attempt to score, rank, and classify daily stressors before assigning chatbots (interventions) to accommodate the differences in low- and high-complexity stressors as well as concerns about identifying problems that are too severe for the system to handle.
4. Consider a multitude of user coping and conversational styles, including users who may need a guided intervention or just an opportunity to reflect by talking or typing it out *into the void*.
5. Measure user personality, chatbot efficacy, and system engagement to optimize interactions across users.

If these problems can be addressed, then there is a real possibility of using this design paradigm to create a new breed of shallow chatbot systems that might be more engaging over the long term. However, the most difficult task is to convey the utility of these shallow chatbots to potential users for daily stress management. For the *Popbots*, our target group is healthy people regularly undergoing daily stress who are less likely to use preventive health systems. These users are a relatively understudied population in mental health, making research into engaging them and allowing them to explore the different interventions—which are available by, for example, using gamification or narrative approaches—an important focus for future research.

### Limitations and Future Work

In addition to the aforementioned items, some additional limitations of this work include that the population in both studies was small and limited to students and staff members of a single university, which is likely not representative of the general population. Moreover, the population consisted largely of women, thus introducing a potential gender skew. The field studies were conducted during a single week, which is not sufficient to capture long-term effects, and, despite its privacy advantages over other platforms, Telegram is not a common messaging app. Downloading this app represents a significant barrier to adoption and may have contributed to attrition (eg, 4/44, 9% of participants registered for the study but did not sign in to Telegram). In addition, the compensation schema used to reduce attrition also incentivized the creation of off-topic data and likely influenced participant behavior. Future work should focus on monitoring and providing feedback about intrinsic improvements and avoid extrinsic incentives.

In the short term, we plan to improve the modularity of our suite design, explore the possibility of adding reminders within the system to improve consistency in use, and implement additional user-experience improvements, including the introduction of new chatbots that explore a larger range of interventions (eg, somatic breathing). To address the limitations of population and timescale in future evaluations, we aim to conduct a randomized controlled trial with a larger sample of diverse participants over a 4- to 8-week period with an appropriate control group and explore additional evaluation metrics that will make comparing the system with others easier (as suggested in the study by Abd-Alrazaq et al [54]). Using the data from this study, we plan to create a web-based learning recommendation system that helps pair users to our *Popbots,* given their stressor and context. An extension of this idea is creating an algorithm to detect whether the *Popbots* can handle a particular stressor and referring users to additional resources if needed (eg, calling 911 or seeking specialized help). To create an ecosystem of support with chatbots, we also plan to develop an authoring tool that will empower both mental health professionals and everyday users to create an increasing number and variety of chatbots, allowing us to compare their performance in numerous ways (eg, with other chatbots and by author type).

### Conclusions

In this study, we have presented *Popbots*—a suite of shallow just-in-time chatbots that help users deal with daily stress. The system is scalable and provides variety in delivering numerous interventions rapidly, preventing attrition. We conducted multiple exploratory studies on the use of these microintervention chatbots for daily stress management, including a WOZ feasibility study, which we used to justify our approach of using multiple chatbots. We then iterated on the design of this system and tested its efficacy in a web-based pilot study. The results indicated that the users experienced a decrease in depression symptoms, viewed conversations as helpful to neutral, and came away with an increasingly positive sentiment toward the use of chatbots for proactive stress management. The follow-up interviews with a subset of the participants indicated that almost half of the common daily stressors could be discussed with chatbots, potentially reducing the burden on human coping resources. In the future, we plan to add new features such as web-based learning recommendation systems while conducting longitudinal studies on the efficacy of the *Popbots* to serve as an effective public health tool.

## Appendix

Multimedia Appendix 1 Additional information about our formative study, examples of conversations with our chatbots, and additional figures.

## Abbreviations

AI: artificial intelligence
CBT: cognitive behavioral therapy
PHQ: Patient Health Questionnaire
WOZ: Wizard of Oz
