# Supplementary material for: A Suite of Mobile Conversational Agents for Daily Stress Management (Popbots): Mixed Methods Exploratory Study
Source: JMIR Form Res. 2021 Sep 14;5(9):e25294. doi: 10.2196/25294 (PMC8479600; doi:10.2196/25294)
Supplement: Multimedia Appendix 1 [file formative_v5i9e25294_app1.docx]

# Multimedia Appendix Popbots—a Suite of Mobile Conversational Agents for Daily Stress Management: Mixed Methods Exploratory Study

## Additional Figures


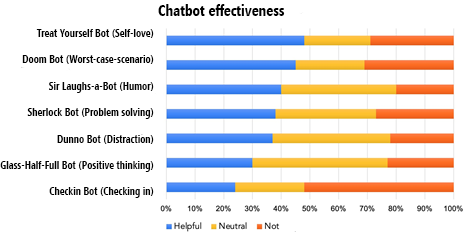


Multimedia Appendix Fig. S1. In situ effectiveness of individual chatbots.

**PHQ scores**


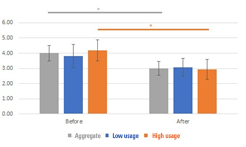


Multimedia Appendix Fig. S2. PHQ-4 scores. PHQ: Patient Health Questionnaire.

**Stress scores**


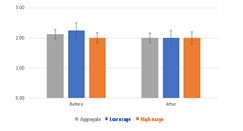


Multimedia Appendix Fig. S3. Stress scores.


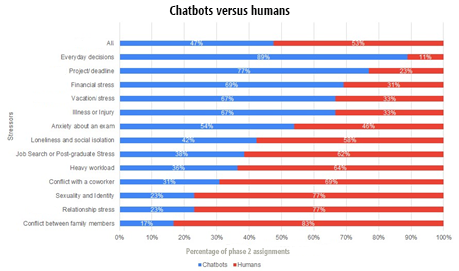


Multimedia Appendix Fig. S4. Stressor assignments by resource in card-sorting task.

## Contents

1. Formative Study 4
   1. Formative Study Protocol 4
   2. Formative Study Results 4
      1. Perceived Stress Reduction 4
      2. Perceptions of Chatbots 4
2. [Additional] References 5
3. Conversation Examples 6
   1. Family Issues 6
   2. Other 6
   3. Health, Fatigue, & Physical Pain 7
   4. Social Relationships 8
   5. Work, School, & Productivity 9

## List of Figures

1. Chatbot effectiveness 1
2. PHQ-4 scores 1
3. Stress scores 2
4. Chatbots versus humans 2
5. Perceived stress reduction 5

## Formative Study

To explore the feasibility of our proposal, we first conducted a formative Wizard of Oz (WoZ) [2] study with follow-up interviews. We recruited N=14 participants (7 male, 6 female, 1 non-binary; age range 18-50) via university listserv. Most (13/14) were university students while one was a staff member. The study lasted 3-days with participants meeting with an experimenter daily for in-person sessions. See [6] for additional details.

#### Formative Study Protocol

We used tlk.io [3], a web chat interface that allows the creation of open chat channels, to serve as the interface between participants and experimenter. Participants believed they were interacting with a chatbot while in reality they were interacting with the experimenter who was following the conversational scripts created in our workshop (*e.g.,* Table 2). Each participant was randomly assigned to either a *Variable* condition that had three chatbots or a *Control* condition that contained only one *(i.e*., problem solving). Participants in the *Variable* condition were matched with different chatbots during each session using Latin Squares Randomization [4]. During each session, participants had a single conversation. The participants were instructed to type a greeting (*e.g.,* “Hi”) in the chat channel which cued the experimenter to start following the script. After 3 sessions, participants completed a post-study questionnaire about perceived efficacy in stress reduction and usability of the chatbots. Four participants were contacted for semi-structured interviews—two from each experiment condition. In each pair, one individual evaluated the chatbot’s effectiveness at the variety of stressors into buckets (*i.e.,* chatbots and their coping strategies) while using a “Think Aloud” protocol to understand trends and underlying motivations. Protocols were reviewed for ethics and privacy concerns by our intuition’s Research Compliance Office.

Formative Study Results

*Perceived Stress Reduction.* When asked about their interactions with the chatbots, analysis of the data shows differences in self-reported stress between conditions (MA Figure 1). Participants reported a higher perception of stress reduction in the *Variable* chatbot condition (blue, left is better), which helps motivate our approach.

*Perceptions of Chatbots.* Overall, participants who completed follow-up interviews were positive about chatbot systems. Most (3/4) were interested in using chatbots for coping with daily stressors even when support from humans was available. The objectivity, ease of use, and privacy chatbots offered compared to human conversational partners were appealing for situations like illness and injury, financial problems, and social isolation. They believed that the chatbots would be effective because they provided quick therapy solutions on the spot. For example, one participant stated, *“I’d rather talk about these [problems] in the void...and have a computer interact with me quickly”.* The other participant preferred to talk to humans. That being stated, all participants expected chatbots to have human-like characteristics (*e.g.,* a typing delay despite being aware that chatbots can respond faster) corroborating prior work on the mirroring of non-verbal [1] and conversational [5] cues. Additionally, one participant from the *Variable* condition described using multiple chatbots (*i.e.,* multiple micro-interventions) in sequence to help with finding appropriate solutions for complex stressors (*e.g.,* using *Positive Thinking* to reduce anxiety first and then *Problem Solving* to take care of the underlying problem) which was not possible in the control condition where participants interacted with a single chatbot (*i.e., Problem Solving*) providing only one intervention.


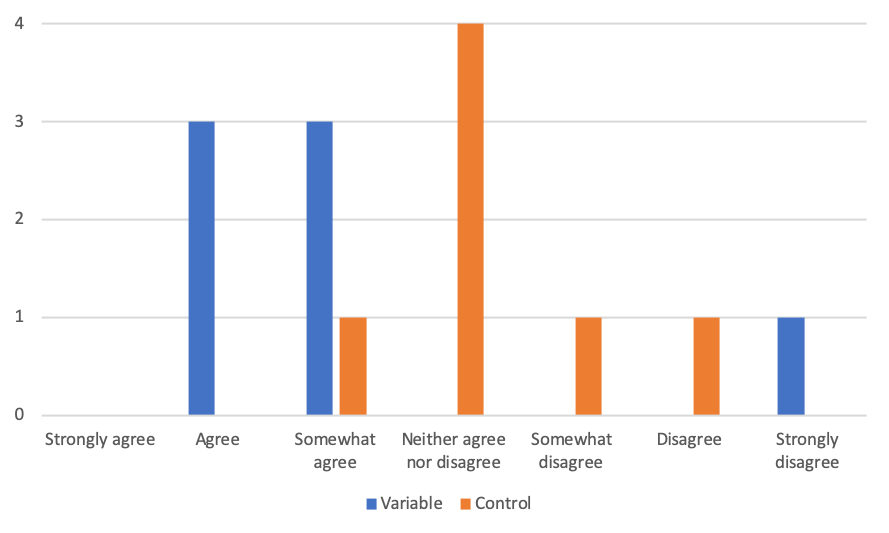


Multimedia Appendix Fig. S5. Participants rated whether they agreed that their interactions with the chatbots had reduced their stress. We observe that participants in the *Variable* condition tended to agree or somewhat agree while those in the *Control* condition were more neutral.

## References

1. Jeremy N Bailenson, Jeremy N Bailenson, Nick Yee, and Nick Yee. 2005. Digital Chameleons. *Society* 16, 10: 814–819. https://doi.org/10.1111/j.1467-9280.2005.01619.x

2. Nils Dahlbäck, Arne Jönsson, and Lars Ahrenberg. 1993. Wizard of Oz studies—why and how. *Knowledge-based systems* 6, 4: 258–266.

3. Anthony Dmitriyev and Simon. 2019. tlk.io. Retrieved September 16, 2019 from https://tlk.io

4. Lei Gao. 2005. Latin Squares in Experimental Design. *Michigan State University*: 1–19. Retrieved from http://www.mth.msu.edu/%7B~%7Djhall/classes/mth880-05/projects/latin.pdf

5. François Mairesse and Marilyn A Walker. 2008. Trainable Generation of Big-Five Personality Styles through Data-driven Parameter Estimation.

6. Pablo E Paredes, Nantanick Tantivasadakarn, Grace Hon, Emmanuel Thierry Lincoln, Nikhil Gowda, Marco A Mora-Mendoza, and Matthew Louis Mauriello. 2020. Towards PopBots: A Suite of Conversational Agents for Daily Stress. 5. Retrieved from http://casforhealth.org/wp-content/uploads/2020/04/Towards-PopBots-A-Suite-of-Conversational-Agents-for-Daily-Stress.pdf

## Conversation Examples

### Family Issues

- Bot: ["Hi Jas. Thanks for texting us again", "What's a recent situation that has been stressing you out?"]
- User: My 13-year-old hates school and tries everything to avoid going
- Bot: ['Hmm... What about this one? (You can always type /switch to change bots again.)']["Hi Jas, I'm **Doom Bot**", 'Tell with me more details about that?']
- User: My daughter had anxiety disorder and Asperger’s. So she has a hard time with the social aspects of school. She gives me and her dad a really difficult time.
- Bot: ['Hmm I see...', 'What are you most worried about happening due to that?']
- User: I’m worried about the stress it puts on her dad and myself. It is also super disruptive to our schedules and jobs
- Bot: ['Thank you for sharing your worry with me', 'Ok, on a scale of 1 to 10, with 1 being almost impossible, how likely is this scenario?']
- User: What do you mean how likely?
- Bot: ['Alright, in the case that this happens, what could you do to get back on track?']
- User: I didn’t even answer your first question.
- Bot: ['Cool, looks like you have a plan B', 'Just remember, even though you cannot control everything, there is a way to get back on your feet', 'Sounds good?']
- User: No. That didn’t make any sense
- Bot: ['Do you think this conversation has helped you to reduce stress? Almost no help, neutral or very helpful?']
- User: Almost no help

### Other

- Bot: ["Hi Matt. Thanks for texting us again", "What's something that's on your mind that is stressing you out?"]
- User: Flat tire
- Bot: ['Hmm... How about this bot?']["Hi Matt, I'm **Glass-half-full Bot**", 'What is it about that that is making you feel stressed?']
- User: No reliable transportation
- Bot: ['I see how that can be stressful', 'I want you to take a couple minutes and think about at least one positive aspect about your situation', 'Then share it with me']
- User: Force me to consider alternate transportation
- Bot: ['Great!', 'Do you think there is another positive alternative you can find in your situation?']
- User: Less mileage on car
- Bot: ["Awesome you're a great positive thinker!!"]["When you're feeling down about a situation, trying to find the positives can make it easier to handle", 'Sounds good?']
- User: Yes
- Bot: ['Do you think this conversation has helped you to reduce stress? Almost no help, neutral or very helpful?']
- User: Helpful

### Health, Fatigue, & Physical Pain

- Bot: ["Hi Samantha. Thanks for texting us again", "What's on your mind that is stressing you out?"]
- User: It's been a long day and I'm looking forward to relaxing
- Bot: ['Which bot do you want to talk to?']
- User: **Sir Laughs Bot**
- Bot: ['Roger! Switching bots!']["Hi Samantha, I'm Sir Laughs Bot", 'Give me more deets about day!']
- User: Cleaned and organized the house and now I want a glass of wine and to relax
- Bot: ['Thank you for sharing', 'That does sound stressful', "Ok, let's try looking at this situation in a different light", 'I want you to take a few minutes to come up with a joke about this situation', ' Would you like an example?']
- User: Sure
- Bot: ['For example, if you are hungry, and you are stuck in traffic', 'This might be a good joke', 'Why do French people eat snails?', "Because they don't like fast food", "Don't worry about it being the best joke, just find something humorous about your situation", 'Share your joke with me!']
- User: Cleaning house with a two-year-old is like being a hamster in a wheel
- Bot: ["Heehee! You're funny!", 'Humor can be found in many situations', 'Okay?']
- User: Yes
- Bot: ["I'm glad. Would you consider trying this strategy, such as finding a joke, in the future?"]
- User: Maybe
- Bot: ['Do you think this conversation has helped you to reduce stress? Almost no help, neutral or very helpful?']
- User: Neutral

### Social Relationships

- User: Hi
- Bot: ["Hi Cassandra. Thanks for texting us again", "What's stressing you out right now?"]
- User: I found out my ex has a new girlfriend
- Bot: ['Which bot do you want to talk to?']
- User: **Glass-half-full Bot**
- Bot: ['Roger! Switching bots! (You can always type /switch to change bots again.)']
- User: Glass-half-full Bot
- Bot: ["Hi Cassandra, I'm Glass-half-full Bot", 'What is it about new girlfriend that is making you feel stressed?']
- User: My ex has moved on and I'm really sad and stressed about my own relationship status
- Bot: ["I'm sorry", 'I want you to try something', 'Take a couple minutes and think about at least one positive aspect about your situation', 'Share it with me when you’re done']
- User: It's good that he's happy.
- Bot: ['Great!', 'Do you think there is another positive alternative you can find in your situation?']
- User: I wish I was happy but I'm not
- Bot: ['Wonderful! See, you can usually find positives even when in the most negative of situations'] ["When you're feeling down about a situation, trying to find the positives can make it easier to handle", 'Sounds good?']
- User: I don’t see a positive right now
- Bot: ['Do you think this conversation has helped you to reduce stress? Almost no help, neutral or very helpful?']
- User: No help

### Work, School, & Productivity

- Bot: ["Hi E. Thanks for texting us again", "What's a recent situation that has been stressing you out?"]
- User: I have some tasks that I keep putting off
- Bot: ['Hmm... What about this one? (You can always type /switch to change bots again.)']
- User: I have some tasks that I keep putting off
- Bot: ["Hi E, I'm **Sherlock Bot**.", 'Give me more details about that.']
- User: They are personal tasks and I have been prioritizing work
- Bot: ["Hmm, let's think about this together", 'Do you feel this problem is significantly affecting your life?']
- User: Yes
- Bot: ['Have you ever dealt with this problem or something similar before?']
- User: Yes
- Bot: ['Cool, tell me about a strategy you used in the past to deal with that similar problem']
- User: Just say to myself okay you need to work on this NOW, and then try to break it up into doable smaller tasks
- Bot: ['Could you adapt or reuse this strategy to solve the current problem?']
- User: I could but haven't yet
- Bot: ['Perhaps breaking down the problem would be helpful', 'Try to describe for me a piece of the problem and a simple solution for it?']
- User: Make contact by phone. Write letter and or fill out forms. Repeat s
- Bot: ["Great, I'm glad you can do something about it", 'By breaking down a problem into tiny steps, we can often string together a solution', 'Sounds good?']
- User: As many times as necessary
- Bot: ['Do you think this conversation has helped you to reduce stress? Almost no help, neutral or very helpful?']
- User: Neutral
